# Improving Postpolypectomy Surveillance Decisions Using an Automated Analysis of Colonoscopy and Histopathology Reports: 2-Center Validation Study

**DOI:** 10.2196/102789

**Published:** 2026-10-16

**Authors:** Agathe Zecevic, Sabina De Martino, Joshua Shan Chen Lee, Victoria To, Vedant Bhardwaj, Rhea Sibal, Nigel Trudgill, Shahd A Mohamed, Angus Roberts, Sebastian Zeki

**Affiliations:** 1 School of Cancer and Pharmaceutical Sciences, Faculty of Life Sciences and Medicine, King's College London, Great Maze Pond, SE1 1UL, United Kingdom, London, United Kingdom; 2 Clinical Scientific Computing, Guy's and St Thomas' NHS Foundation Trust, London, United Kingdom; 3 Gastroenterology Department, Guy's and St Thomas' NHS Foundation Trust, Gassiot House, St Thomas' Hospital, Westminster Bridge Road, London, SE1 7EH, United Kingdom; 4 GKT School of Medical Education, Faculty of Life Sciences and Medicine, King's College London, London, United Kingdom; 5 Gastroenterology Department, Sandwell and West Birmingham Hospitals NHS Trust, West Bromwich, United Kingdom; 6 Department of Cancer and Genomic Sciences, University of Birmingham, Birmingham, United Kingdom; 7 Biostatistics and Health Informatics, Institute of Psychiatry, Psychology and Neuroscience, King's College London, London, United Kingdom

**Keywords:** colorectal polyps, clinical decision support, natural language processing, large language model, gastroenterology, endoscopy, artificial intelligence, AI

## Abstract

**Background:**

Postpolypectomy surveillance intervals derived from colonoscopy findings and histopathology should be guideline-based, yet these are frequently not adhered to, contributing to under- and oversurveillance. This motivates a more structured and automated approach to improve adherence.

**Objective:**

We developed and evaluated an automated, implementable, guideline-aligned decision support tool that extracts colorectal polyp features from free-text colonoscopy and histopathology reports and produces auditable surveillance recommendations.

**Methods:**

We extracted paired colonoscopy and histopathology reports from a National Health Service (NHS) trust and designed a 4-stage prompting pipeline using open-weight large language models to separate multispecimen histopathology reports into individual containers (“pots”), align endoscopic findings with specimens, and normalize each pot into a structured schema. The surveillance intervals were then computed by a deterministic rule engine encoding guideline rules. We evaluated extraction performance on 100 procedures (215 pots), assessed surveillance recommendation performance on a same-site cohort (n=100), and performed external validation on a cohort from a second trust (n=100). A separate retrospective audit compared automated recommendations and observed routine scheduling against a clinician-derived reference based on full electronic health record review in 127 patients.

**Results:**

Key extraction fields showed end-to-end accuracy (specimen type=0.995; location=0.981; diagnosis=0.981; excision completeness=0.949), with mean report-level pot comprehensiveness of 0.968 (SD 0.112). Overall surveillance recommendation accuracy was 0.96 (95% CI 0.90-0.98) at Guy’s and St Thomas’ NHS Foundation Trust (GSTT) and 0.93 (95% CI 0.86-0.97) at Sandwell and West Birmingham NHS Trust (SWB) and showed strong class-level performance (no follow-up *F*_1_-score of 0.98 at GSTT and 0.97 at SWB; site check *F*_1_-score of 0.93 at GSTT and 0.91 at SWB; 3-year follow-up *F*_1_-score of 0.86 at GSTT and 0.80 at SWB; cancer alert *F*_1_-score of 1.00 at GSTT and 1.00 at SWB). The comparison to current clinical practice showed that the tool would improve guideline adherence primarily by reducing potential undersurveillance.

**Conclusions:**

This hybrid approach using open-weight large language models for structured extraction and guideline-encoded rules for decision-making can generate accurate, lightweight, and auditable postpolypectomy surveillance recommendations. Such a tool has the potential to improve safety netting and guideline adherence in NHS colonoscopy workflows.

## Introduction

### Background

Colorectal polyps are a significant risk factor for colorectal cancer (CRC). When polyps are identified during an index colonoscopy, patients may require further colonoscopic surveillance depending on the endoscopic and pathological characteristics of the lesions [1]. Colorectal polyp surveillance improves the detection of CRCs, particularly in high-risk groups defined by specific endoscopic and pathological findings [2-5]. Endoscopy represents a substantial workload in the United Kingdom, with around 2 million procedures performed each year. Polyp surveillance accounts for approximately 15% of the half a million colonoscopies performed annually [1,6].

Postpolypectomy surveillance decisions are particularly vulnerable to inaccuracies. Reported compliance with the updated 2020 postpolypectomy surveillance guidelines varies from 66.2% to 86.5% [7,8]. Poor compliance can often be attributed to surveillance intervals not being updated once pathology results become available [9]. Nonadherence to guidelines can lead to both under- and oversurveillance, with one study reporting 73% of nonadherent patients having earlier-than-recommended colonoscopies [9]. These findings highlight the need for continued efforts to improve guideline adherence in colorectal surveillance and the need for accurate calculation of surveillance intervals based on evolving guidelines.

Several studies have proposed to automate portions of the decision-making workflow, but they frequently rely on applying inflexible rules or pattern recognition to endoscopy and pathology report text (regular expressions) that struggle to accommodate variations in clinical report language. Existing studies are often limited to single-center data or focus on partial tasks such as adenoma detection without extending to full guideline-based follow-up recommendations [10-13]. Wu et al [14] developed an automatic surveillance system for postpolypectomy follow-up that encodes multiple international guidelines and combines free-text endoscopy and pathology reports to assign surveillance intervals. Their approach relies heavily on regular expressions, which are used to identify patients and map extracted polyp attributes. In practice, this design requires a long list of exclusion categories (no pathology result; poor bowel preparation; previous CRC, colorectal surgery, or endoscopic submucosal dissection; nonpolyp lesions; hamartomas; inconsistent endoscopic vs pathological reports; and missing or malformed size information) so that many real-world reports fall back to manual review rather than fully automated surveillance. The system was developed and evaluated in three hospitals in China, which may limit direct transferability to English-language histopathology workflows in UK practice.

Recent studies have evaluated general-purpose large language models (LLMs) for postpolypectomy surveillance interval prediction [15-17]. The larger study by Acharya et al [17] leveraged GPT-4o to generate recommendations from colonoscopy and pathology report impressions using prompted US Multi-Society Task Force guidance. These approaches treat surveillance interval generation as an end-to-end language model task, which leaves clinical decision-making implicit without auditable intermediate representation for clinicians to review.

Our study used a different task breakdown: explicit extraction of specimen-level variables followed by separately implemented British Society of Gastroenterology (BSG) guideline rules. This approach allows for evaluation of the extracted variables and the final interval suggestion. This also allows decision rules to be modified alongside guideline changes independently of the language model. Additionally, we compared automated recommendations with observed care under real-world deployment conditions, looking at patients’ full electronic health record (EHR) history beyond the index colonoscopy.

### Aims

The primary objective of the current study was to develop and retrospectively evaluate an implementable and interpretable hybrid decision-making tool that accurately suggests follow-up timings for patients undergoing colorectal surveillance based on colonoscopy and associated histopathology reports grounded in BSG guidelines. We validated our tool on reports from two geographically disparate National Health Service (NHS) trusts in the United Kingdom (Guy’s and St Thomas’ NHS Foundation Trust [GSTT] and Sandwell and West Birmingham NHS Trust [SWB]).

A secondary objective was to assess the tool’s potential clinical utility in routine NHS care. We compared automated recommendations with observed surveillance timing provided by gastroenterologists’ review of full EHRs. This approach enabled us to understand whether automated decision support could reduce under- or oversurveillance and identify limitations of decisions based on the index reports.

## Methods

### Overview

Figure 1 summarizes the entire pipeline from data access to the evaluation of the AI component. We adhered to the TRIPOD-LLM (Transparent Reporting of a Multivariable Prediction Model for Individual Prognosis or Diagnosis–Large Language Models) guidelines [18] and completed the checklist (Checklist 1) ().

**Figure 1.**
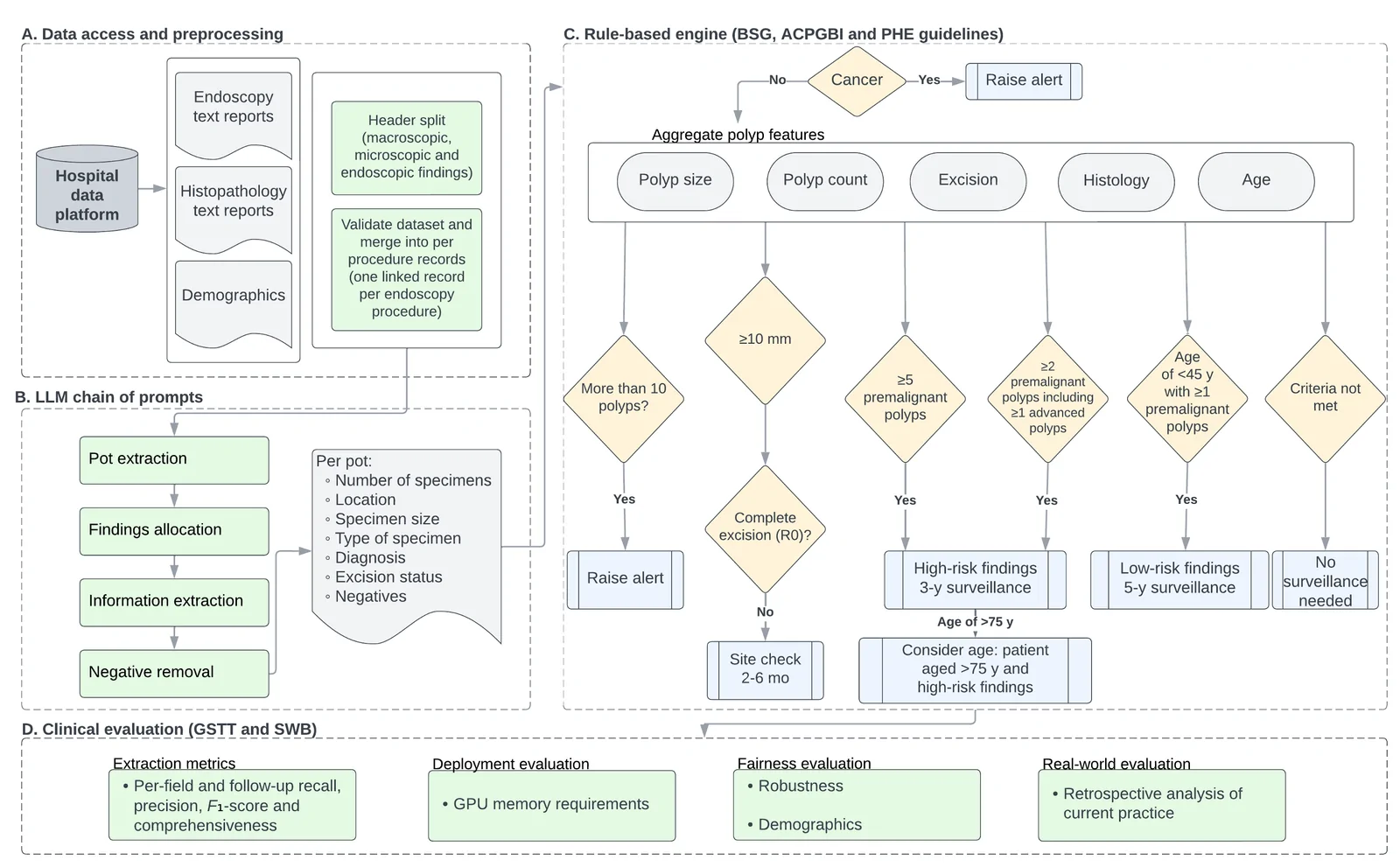
Overview of the entire methodology, including data access (A), large language model (LLM) information extraction (B), guideline-grounded rule-based algorithm (C), and multi-trust evaluation (D). ACPGBI: Association of Coloproctology of Great Britain and Ireland; BSG: British Society of Gastroenterology; GPU: graphics processing unit; GSTT: Guy’s and St Thomas’ National Health Service Foundation Trust; PHE: Public Health England; SWB: Sandwell and West Birmingham National Health Service Trust.

### Ethical Considerations

Data access and processing at GSTT was authorized by the Guy’s and St Thomas’ Electronic Records Research Interface (Integrated Research Application System [IRAS] ID 257283; Research Ethics Committee ID 20/EM/0112). This approval was effective from June 3, 2023. Data access for SWB was authorized through a data sharing agreement between the institutions in compliance with the General Data Protection Regulation. This work used deidentified, routinely collected clinical data with no direct participant contact. Individual informed consent was not required under the governance and ethics approvals for the data access route. Patients in England can register in the National Data Opt-Out service to prevent use of their confidential patient information for purposes beyond individual care (research and planning).

### Data Sources

We extracted colonoscopy and histopathology text reports from GSTT’s EHR system covering procedures performed between January 2017 and September 2023.

#### Inclusion Criteria

Colonoscopy reports with the mention of the keywords “polyp” or “lesion” were extracted. Corresponding histopathology reports were merged on a per-procedure basis using the *EndoMineR* package [19] by patient identifier and fuzzy-matched date, resulting in a dataset of over 14,735 reports and 11,538 unique patients. Cases with no pathology information were excluded. Patients under the age of 18 years and patients who had opted out of data analysis using the NHS opt-out system were also excluded. The latter program allows individuals to prevent their confidential patient information from being used for research and planning purposes [20]. The final reports were anonymized by removing all personally identifiable information, including patient names, addresses, and phone numbers. The dataset only contained essential clinical fields for decision-making: endoscopic findings (endoscopy report), macroscopic description (histopathology report), microscopic description (histopathology report), and date of birth. More details about patient involvement can be found in Multimedia Appendix 1.

#### Training and Test Sets

Ten GSTT reports spanning a range of complexities were selected for model development (in-context learning). For evaluation, we randomly sampled 100 GSTT procedures from unique patients, corresponding to 215 individual histopathology specimen pots from the same date range, excluding the reports included for in-context learning. The test set size was defined as a pragmatic internal validation size given the time required for gold-standard annotations. Table 1 summarizes the demographics of the GSTT test set. For the comparison with current clinical practice, we extracted 127 consecutive GSTT colonoscopy procedures performed between January 2021 and September 2021, along with their associated histopathology reports.

**Table 1.** Demographic characteristics of the Guy’s and St Thomas’ National Health Service Foundation Trust (GSTT) test set compared with the full GSTT cohort.

Characteristics	GSTT test set (n=100)	GSTT original cohort (n=14,735)
Sex, n (%)		
Female	45 (45)	6722 (45.6)
Male	55 (55)	8012 (54.4)
Other or unknown	0 (0)	1 (0.0)
Ethnicity, n (%)		
Asian	2 (2)	608 (4.1)
Black	2 (2)	1892 (12.8)
White	93 (93)	7625 (51.7)
Other or not specified	3 (3)	4610 (31.3)
Age (y), mean (SD; range)	69.4 (9.35; 48-86)	57.3 (14.50; 18-94)

#### SWB External Validation Cohort

An independent set of 100 colonoscopy and histopathology text reports was obtained from SWB. The same inclusion criteria and deidentification procedures were applied. Date of birth was not shared for the SWB cohort. A CONSORT (Consolidated Standards of Reporting Trials) diagram describing the datasets is provided in Figure 2.

**Figure 2.**
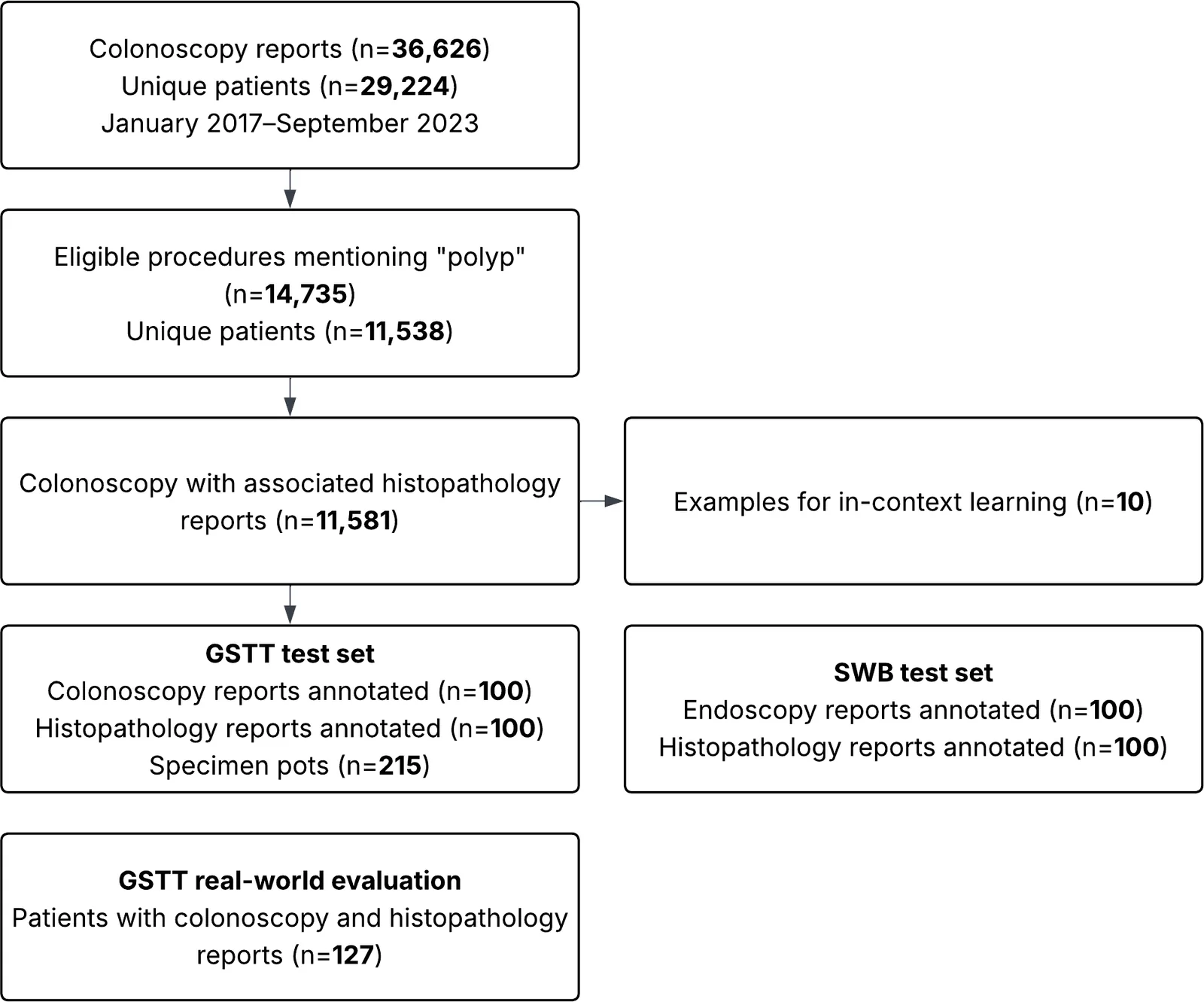
Cohort description. GSTT: Guy’s and St Thomas’ National Health Service Foundation Trust; SWB: Sandwell and West Birmingham National Health Service Trust.

#### Data Governance and Approvals

As mentioned, data acquisition at GSTT was authorized through institutional board review (IRAS ID257283), under the governance of the Guy’s and St Thomas’ Electronic Research Records Interface. SWB data was provided under a data sharing agreement between institutions. No identifiable patient information left any of the NHS trusts. Patient age was not shared for the SWB cohort, making the algorithm bypass age-dependent conditions.

### Task Definition and Annotation

The pipeline aims to extract guideline-relevant polyp features from paired colonoscopy and histopathology reports and then apply the BSG guidelines to generate a follow-up recommendation. A key challenge is that the evidence needed for surveillance is fragmented across sections: endoscopic findings describe the lesions sampled, whereas histopathology reports describe multiple specimens (“pots”) with macroscopic and microscopic text that must be linked to determine which pots correspond to polyps, which represent nonpolyp tissue, and which findings are unresected or described only endoscopically. The GSTT test set was annotated by a committee of 3 clinical experts to establish the gold standard. For each procedure, annotators reviewed the endoscopic findings and the histopathology macroscopic and microscopic descriptions to derive the number of histology pots and extract pot-level variables listed in Table 2. “Premalignant” and “advanced” polyps were defined according to the BSG criteria [1]. Disagreements between annotators were resolved through consensus. When consensus could not be reached, a fourth clinical expert (SZ) resolved the discrepancy. The SWB test set was annotated by a single gastroenterologist (SZ).

**Table 2.** Information extraction annotation categories and definitions.

Category	Definition
Number of pots	Total number of histology pots submitted for a given colonoscopy
Specimen type	Classification of the tissue sample (polyp, biopsy, mucosal fragment, etc)
Size	Dimensions of all specimens within the pot
Number of specimens—findings	Number of polyps or biopsies documented in the colonoscopy report
Number of specimens—macro	Number of specimens received as per the histopathology macroscopic description
Location	Anatomical site of origin (such as rectum or ascending, descending, or sigmoid colon)
Diagnosis	Extracted histopathological diagnosis (such as tubular adenoma or high-grade dysplasia)
Other polyp characteristics	Details of polyps described endoscopically but not resected or sampled
Excision completeness	Completeness of excision: “yes” (R0 or en bloc) or “no” (piecemeal or uncertain)
Negation	Presence of negated diagnostic terms (“no adenocarcinoma”)

### Approach and Model Selection

#### Overview

Our objective required extracting multiple interdependent variables (Table 2) from heterogeneous free-text colonoscopy and histopathology reports. Pot-level alignment and cross-referencing were difficult to model reliably using simple pattern-matching rules. A purely supervised approach would require large-scale, pot-level annotations to generalize across report templates and anatomical sites. In preliminary experiments, a fine-tuned encoder-based baseline did not generalize well under the available annotation budget. We therefore used LLMs as flexible extractors that could be constrained to produce structured outputs with minimal labeled data. The design of this study explicitly separates information extraction from clinical decision-making. The LLM was not used to infer risk categories or recommend intervals. The final surveillance recommendation was computed by a deterministic, auditable, guideline-encoded rule engine.

#### Model Selection

Given typical on-premises hospital hardware constraints, we evaluated which open-weight instruction-tuned LLM size was sufficient for this task without additional supervised fine-tuning. We compared multiple models (Microsoft Phi-4, Phi-4-mini-instruct, Llama 3.1-8B-Instruct [Meta AI], Falcon3-7B-Instruct [Technology Innovation Institute], and Mistral-7B-Instruct-v0.3 [Mistral AI SAS]) under an identical prompting, decoding, and parsing configuration. We report the surveillance accuracy for each model in Multimedia Appendix 2. We used Microsoft Phi-4 [21] (Hugging Face) at revision 932b33c for the actual pipeline.

#### Hardware and Pipeline Setup

All LLM inference was executed on local graphics processing unit (GPU) hardware (NVIDIA DGX-1 server running Ubuntu 20.04, configured with eight 32-GB V100 GPUs and 512 GB of RAM). We loaded the model with 4-bit quantization and initialized the model with automatic sharding across available GPUs. Generation was performed in batches to improve throughput. Decoding was deterministic, with a temperature set to 0. Each pipeline step used a configured maximum number of new token budget. When output parsing failed, the pipeline retried generation with an increased token budget per attempt.

#### Multi-Prompt Strategy

Breaking down complex information extraction tasks into smaller subtasks has proven to improve the performance of LLM pipelines [22]. The task at hand was complex by nature as relevant information is fragmented across report sections and multiple specimens (pots) must be aligned before guideline features can be extracted. We therefore broke down the pipeline into four sequential prompts that progressively (1) segmented histopathology text pot by pot, (2) appended endoscopic findings to the corresponding pots, (3) normalized each pot into a structured schema, and (4) extracted guideline-relevant attributes. Figure 3 illustrates this progressive transformation for a synthetic report.

**Figure 3.**
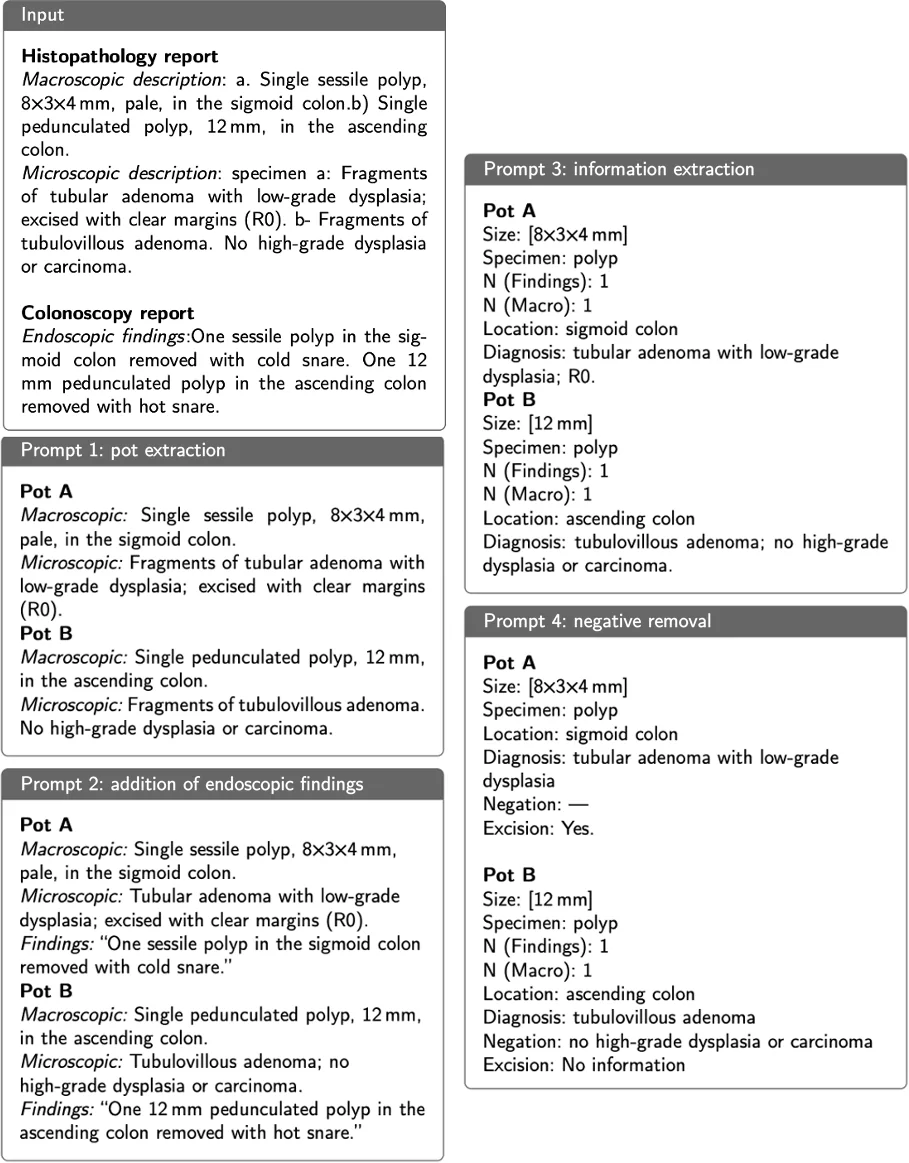
Description of the information extraction pipeline and example of the evolution of an input colonoscopy and histopathology report, prompt by prompt.

### Rule-Based Logic

The structured outputs produced by the final LLM prompt were parsed into histopathology pot-level variables (Table 2) and passed to a deterministic rule-based algorithm that encoded the guidelines. The algorithm first raised an alert if cancer was detected, then filtered out nonpolyp specimens; aggregated polyp counts and sizes across all pots; identified premalignant and advanced polyps using guideline-aligned criteria; and, finally, output a surveillance recommendation according to the BSG, Association of Coloproctology of Great Britain and Ireland, or Public Health England risk categories (no surveillance, 3-year surveillance, 5-year surveillance, or site check for large nonpedunculated polyps with uncertain excision). If age was missing, the algorithm ignored age-based conditions and defaulted to the non–age-specific guideline branches.

The pseudocode functions “IsPremalignantPolyp,” “IsAdvancedPolyp,” and “IsHighRisk” implemented guideline definitions of premalignant polyps, advanced polyps, and high-risk patients. In summary, premalignant polyps included adenomatous polyps and clinically relevant serrated lesions (excluding diminutive rectal hyperplastic polyps), and advanced polyps were defined by size and/or dysplasia according to the UK guideline thresholds. High risk was assigned when either 2 or more premalignant polyps were present, including 1 or more advanced polyps, or 5 or more premalignant polyps were present.

The algorithm compared the endoscopic polyp count with the number of histopathology specimens to detect specimen fragmentation and reduce pot-level size and count estimation bias. The algorithm preferentially used the endoscopic size as pathology-reported measurements may underestimate true size due to specimen shrinkage after formalin fixation [23]. The pseudocode for the rule-based algorithm can be found in Multimedia Appendix 3.

### Model Evaluation

#### Overview

Most studies on LLMs in health care focus on accuracy. However, accuracy alone is not enough for clinical integration: reliability, completeness, and deployment feasibility are equally important. We evaluated our chain-prompting pipeline across multiple dimensions aligned with best practices in health care LLM evaluation [24-26]. Some commonly used evaluation criteria such as human preference do not apply to this study, which is focused on structured information extraction rather than free-text generation.

#### Accuracy and Comprehensiveness

Accuracy was assessed by comparing the model’s extracted values to gold-standard annotations for each category. We evaluated information extraction at the pot level by comparing the model output against clinician gold-standard annotations. For single-value fields, including number of pots, specimen type, excision completeness, location, number of specimens—findings, and number of specimens—macroscopy, correctness was defined as an exact match to the gold-standard label. For the size field, order was ignored, but all size entries had to match the gold-standard annotations. Diagnosis was evaluated using a structured “diagnosis signature” derived from the microscopic description consisting of information about the lesion type (tubular adenoma, tubulovillous adenoma, serrated lesion, or hyperplastic), dysplasia grade (none, low grade, or high grade), and invasive cancer (present or absent). A diagnosis was scored as correct only if all components of the signature matched the gold-standard annotation for that pot. Negation was evaluated by comparing the set of clinically critical negated concepts extracted for each pot to the gold-standard set.

Comprehensiveness was evaluated at the report level as the proportion of gold-standard fields whose values were extracted exactly correctly. Because incorrect or missing values are counted as false negatives, this metric is equivalent to per-report, field-level recall:

Comprehensiveness = (number of gold-standard fields extracted exactly correctly)/(number of gold-standard fields)

We averaged this score across reports to obtain an overall measure of the model’s ability to extract all the expected fields. We also report the *F*_1_-score per category, capturing both precision and recall. The 95% Wilson CIs were calculated for overall recommendation accuracy and class-specific precision and recall.

#### Robustness

Robustness was assessed by evaluating the model’s performance on a perturbed version of the test set, inspired by established methods in the literature [27-29]. To simulate real-world typos, we introduced controlled typographical noise, including adjacent letter swaps, random character deletions, insertions, random word splits, and letter case alterations. Synonym replacement was not performed as it is rarely encountered in real-world practice (eg, “adenoma” has no exact lexical variant). Key terms such as “polyp,” “adenoma,” and “dysplasia” were intentionally corrupted at least once per noisy test set to test the model’s resilience to critical colorectal polyp terminology distortions. We generated multiple noisy test sets with increasing perturbation levels (1 to 5 per report) and compared their *F*_1_-scores against that of the original clean set. Examples can be found in Multimedia Appendix 4.

#### Deployment Feasibility

Deployment feasibility was assessed by measuring computational requirements, including the number of model parameters and memory use.

### Retrospective Analysis of Current Practice

In addition to per-report extraction metrics, we performed a retrospective, real-world eligibility study where we compared the automated system recommendations and observed care to a clinician-derived reference based on full EHR review. The automated system used the index colonoscopy and associated histopathology reports, with age where available. This analysis aimed to evaluate how the automated surveillance recommendations would align with guideline-based care when full longitudinal patient information was available.

A single gastroenterologist (SDM) reviewed the complete EHR of all the patients in the retrospective test set (n=127). The clinician determined the gold-standard guideline-concordant surveillance interval for each patient accounting for but not limited to previous CRC or colorectal surgery, prior large or advanced polyps, hereditary or other high-risk conditions, and comorbidities or contraindications that would alter surveillance timing. We then compared 3 intervals: the clinician gold-standard interval from full EHR review, the interval recommended by the automated system using only the index colonoscopy and associated histopathology reports, and the observed care interval based on real-world scheduling (a completed follow-up colonoscopy or a future booking recorded in the EHR). Observed care was assessed using all information available in Epic. A completed procedure, future booking, or absence of surveillance were classified looking at all post–index colonoscopy data available in Epic. Reference assessment was blinded to model output. This analysis assessed the extent to which this system could improve alignment with guideline-concordant surveillance and, therefore, its potential impact on clinical practice. Full EHR review also identified patients whose management required a clinical pathway beyond the postpolypectomy recommendations generated by the system.

## Results

We report per-field extraction performance at GSTT, surveillance recommendation performance at GSTT and SWB, robustness to typographical noise, an analysis of the trade-off between model size, and on-premises feasibility and performance. Unless otherwise stated, results correspond to the final Phi-4 multistage prompting configuration.

### Extraction Performance

Table 3 shows that the extraction of key fields for the final surveillance decision was highly reliable (mean comprehensiveness 0.968, SD 0.112), with close to perfect accuracy for location, type of specimen, and histopathological size (0.981, 0.995, and 0.995, respectively). The model also showed high accuracy for diagnosis (0.981) and excision completeness (0.949). The endoscopic size, number of specimens, and extraction of unresected polyps showed the lowest accuracy (0.935, 0.935, and 0.903, respectively).

**Table 3.** Per-field extraction performance.^a^

Extracted fields	Precision	Recall	*F*_1_-score	Accuracy	Support^b^
Type of specimen	1.000	0.995	0.998	0.995	215
Size—findings	0.939	0.925	0.932	0.935	133
Size—macro	1.000	0.995	0.998	0.995	215
Number of specimens—findings	0.945	0.957	0.951	0.935	162
Number of specimens—macro	0.962	0.958	0.960	0.958	214
Location	0.986	0.981	0.984	0.981	215
Diagnosis	0.981	0.981	0.981	0.981	215
Excision completeness	0.958	0.949	0.953	0.949	215
Negation	0.995	0.990	0.992	0.991	193
Other polyp characteristics^c^	0.709	0.938	0.808	0.903	65

a Precision, recall, and *F*_1_-score were computed using exact-match entity extraction. Accuracy is the overall end-to-end accuracy: correct extraction when the ground truth was present or correctly outputting nothing when the ground truth was absent. Comprehensiveness is the proportion of gold-standard fields extracted exactly correctly for each report (mean pot comprehensiveness 0.968, SD 0.112; mean other polyp comprehensiveness 0.920, SD 0.277).

b Support corresponds to the number of pots where the category is present.

c Microaveraged over other fields (location, number of specimens—findings, and size—findings).

### Surveillance Recommendation Performance and Cross-Site Generalization

Table 4 summarizes the performance of the decision support system for surveillance recommendations at GSTT and SWB. The system achieved an overall accuracy of 0.96 at GSTT and 0.93 at SWB, with strong performance for site check (*F*_1_-score of 0.93 at GSTT and 0.91 at SWB) and no follow-up (*F*_1_-score of 0.98 at GSTT and 0.97 at SWB). Cancer alerting exhibited perfect performance (*F*_1_-score=1.00) for both sites.

**Table 4.** Performance of the decision support system for surveillance recommendations at 2 National Health Service (NHS) trusts (Guy’s and St Thomas’ NHS Foundation Trust [GSTT] and Sandwell and West Birmingham NHS Trust [SWB]).^a^

Categories and metrics	GSTT		SWB	
	Score	Support^b^	Score	Support^b^
Site check		23		35
Precision (95% CI)	1.00 (0.84-1.00)		0.91 (0.78-0.97)	
Recall (95% CI)	0.87 (0.68-0.95)		0.91 (0.78-0.97)	
*F*_1_-score	0.93		0.91	
3-y follow-up		9		12
Precision (95% CI)	0.75 (0.47-0.91)		0.77 (0.50-0.92)	
Recall (95% CI)	1.00 (0.70-1.00)		0.83 (0.55-0.95)	
*F*_1_-score	0.86		0.80	
No follow-up		63		50
Precision (95% CI)	0.98 (0.92-1.00)		0.98 (0.89-1.00)	
Recall (95% CI)	0.98 (0.92-1.00)		0.96 (0.87-0.99)	
*F*_1_-score	0.98		0.97	
ALERT (CRC^c^ or >10 polyps)		5		3
Precision (95% CI)	1.00 (0.57-1.00)		1.00 (0.44-1.00)	
Recall (95% CI)	1.00 (0.57-1.00)		1.00 (0.44-1.00)	
*F*_1_-score	1.00		1.00	
Overall accuracy (95% CI)	0.96 (0.90-0.98)	*100*	*0.93* (0.86-0.97)	*100*

a Wilson 95% CIs are shown for overall accuracy and class-specific precision and recall. The pipeline was run 5 times with different seeds and observed identical outputs across runs (run-to-run SD 0).

b Support corresponds to the number of reports where the category is present.

c CRC: colorectal cancer.

### Robustness

Detailed robustness results across perturbation levels are provided in Multimedia Appendix 5. Across progressively perturbed test sets (1 to 5 perturbations per report), *F*_1_-scores decreased only modestly compared with the clean test set, indicating that the pipeline is robust to realistic typographical noise and minor formatting artifacts. In particular, performance on the number of pots, type of specimen, and diagnosis remained stable, whereas size extraction showed the greatest sensitivity to perturbations.

### Retrospective Analysis of Current Practice

Using gold-standard guideline-concordant intervals derived from full chart review, we audited current practice within the department for 127 patients in the test cohort and compared it to the system’s suggestions. Overall, only 59.1% (75/127) of patients were booked in line with the recommended surveillance interval. A further 15.7% (20/127) deviated from guideline-concordant care: 5.5% (7/127) were potentially oversurveilled (either booked earlier than recommended or scheduled for an unnecessary colonoscopy), and 10.2% (13/127) were potentially undersurveilled (6/127, 4.7% were booked later than recommended, and 7/127, 5.5% had no surveillance booked despite needing one). A further 25.2% (32/127) were managed via alternative pathways (CRC or other colonoscopy pathways), where surveillance scheduling was driven by additional clinical findings (Table 5). Even excluding these alternative-pathway cases, 21.1% (20/95) of patients in routine care had potentially missed or unnecessary surveillance relative to guideline-concordant recommendations. In contrast, our system provided a consistent, automated, and guideline-based recommendation for every patient based on structured information from index colonoscopy and histopathology reports. Overall, the decision support tool improves alignment with guideline-concordant care (Table 5), particularly by reducing undersurveillance. These findings highlight a real opportunity to identify patients at risk of undersurveillance and potentially reduce unnecessary procedures, supporting efficient use of endoscopic resources.

**Table 5.** Comparison of the automated decision support system with current hospital practice (clinician-led booking) using clinician-derived ground-truth intervals from full chart review (n=127).

Interval relative to clinician reference interval	Decision support system, n^a^	Clinician-led booking, n^a^
Concordant with reference interval (subtotal)^b^	88	75
No surveillance needed	65	63
Cancer or ALERT	6	6
Site check at 2-6 mo	7	2
3-y surveillance	7	4
5-y surveillance	3	0
Age of >75 y with high-risk findings	0	0
Potential oversurveillance (subtotal)	4	7
Interval earlier than guideline	1	2
Unnecessary surveillance	3	5
Potential undersurveillance (subtotal)	3	13
Interval later than guideline	1	6
No surveillance booked despite indication	2	7
Total excluded	32	32

a n corresponds to a single patient in this case.

b Guideline-concordant surveillance interval determined by a gastroenterologist from full electronic health record review.

## Discussion

### Principal Findings

This study introduced a decision support tool that combines open-weight, medium-sized language models for extraction of guideline-relevant information with a deterministic rule-based algorithm for postpolypectomy recommendations. Focusing on implementation encouraged the use of a computationally lightweight LLM to only perform report standardization and information extraction. We demonstrated that such a decision aid system presents an opportunity to improve guideline adherence by conducting a retrospective chart review using patients’ full medical histories, showing that routine surveillance sometimes occurs later than guideline-recommended intervals. We identified cases with no indicated surveillance booking, highlighting a concrete opportunity to strengthen safety netting and reduce missed surveillance in routine care.

Acharya et al [16,17] evaluated direct LLM-based surveillance interval assignment in an initial 100-case feasibility study and subsequently in a 2-institution cohort of 1000 cases. The latter study reported a mean GPT-4o accuracy of 94.6% across 10 runs. Our recommendation accuracy was 96% at GSTT (95% CI 90%-98%) and 93% at SWB (95% CI 86%-97%). These independently obtained results cannot be compared directly as the studies differ in guideline systems, populations, output categories, and evaluation scale. The later study by Acharya et al [17] also provides evidence from a substantially larger validation cohort.

In the larger study by Acharya et al [17], accuracy was lower for cases containing 4 or more polyps than for those with 1 to 3 polyps. This finding highlights the importance of evaluating complex cases. Our framework separates specimen-level extraction and aggregation from deterministic guideline application, which facilitates granular evaluation at the individual polyp level.

### Strengths

There are several key strengths to this study. While general-purpose LLMs are increasingly used for a wide range of tasks, our work focuses on a specialist clinical setting in which smaller open-weight models are applied to narrow, auditable subtasks. This design choice supports transparency and increases clinician confidence by making the extracted information and decision logic available for inspection. We divided the overall task into components that closely mirrored clinical practice: collating endoscopy and histopathology reports, extracting information pot by pot, aggregating findings across pots, and deriving a surveillance recommendation. It is important to highlight that the language model was only tasked with extracting and normalizing information already present in the reports and did not generate the surveillance recommendation. The LLM did not generate any clinical decision-making, and the surveillance decision itself was made by a rule-based algorithm anchored in the guidelines, which can be audited and updated independently as guidelines change. By separating extraction from decision-making, the framework could also be adapted to other gastrointestinal surveillance pathways by modifying the extraction layer and introducing condition-specific rules.

The evaluation of the language model information extraction was performed using a range of metrics (accuracy, comprehensiveness, *F*_1_-score, robustness, and deployment considerations) that provide a more complete picture of performance than accuracy or *F*_1_-score alone. The SWB evaluation provided evidence of performance in a second environment with differing documentation styles and reporting practices subject to its small sample size and the restriction concerning age-dependent rules.

This study deliberately focused on small- to medium-sized language models to reflect typical on-premises hardware constraints in NHS settings. While some hospitals can process clinical text via external LLM APIs, this capability varies widely across institutions due to governance and privacy requirements, and on-premises inference is generally preferred.

### Limitations

Future work should include larger and more geographically diverse sites to better assess robustness and generalizability.

The retrospective analysis highlighted that a subset of patients (n=32) required referral to alternative pathways (eg, inflammatory bowel disease or ulcerative colitis), which is currently out of scope for this study. This reinforces why this study introduced a decision support tool rather than a fully automated system: clinicians sometimes need to combine multiple guideline pathways and clinical context to make the correct decision. The SWB and the retrospective audit annotations were provided by a single gastroenterologist, which may introduce reference standard bias.

The two 100-procedure test sets provided limited precision, particularly for minority classes. Larger cohorts with diverse representations of rare outcomes are needed to draw broader generalizability conclusions. The small number of ALERT cases resulted in wide CIs.

The GSTT test set was older (mean 69.4, SD 9.35 vs 57.3, SD 14.50 years) and had a higher recorded proportion of procedures involving White patients (93/100, 93% vs 7625/14,735, 51.7%). Interpretation was also affected by the larger combined “other or not specified” category in the source cohort (4610/14,735, 31.3% vs 3/100, 3%). These differences limit representativeness and may reflect differences in the sampled clinical population. The small numbers in some demographic groups precluded formal subgroup performance analyses across sex or ethnicity. Instead, we report overall performance and highlight fairness as an important direction for future work, particularly as larger and more diverse validation cohorts become available.

Finally, although we compared models of different sizes, the final configuration used a 14 billion–parameter model with 4-bit quantization on the reported hospital server. While our institution has the necessary infrastructure, we acknowledge that this may not be the case in other settings. However, the modular design and code available upon request allow the language model component to be swapped for smaller or fine-tuned alternatives, enabling adaptation to local computation constraints.

### Conclusions

This study demonstrates that a hybrid automated system can generate accurate and auditable postpolypectomy surveillance recommendations from paired colonoscopy and histopathology free-text reports. The framework enables inspection of intermediate information and rule application. External validation across two NHS trusts suggests that the approach could generalize across different reporting environments. The retrospective comparison with routine care suggests potential improvements over current surveillance practice in specific patient groups. It also identified 25.2% (32/127) of patients requiring alternative clinical pathways, highlighting the importance of broader patient history. Future research could therefore investigate whether integrating longitudinal clinical information helps distinguish patients eligible for routine postpolypectomy surveillance from those requiring alternative pathways. Future research could also explore whether smaller open-weight language models can be used to extend this framework and more broadly support gastroenterology surveillance pathways under realistic deployment constraints.

## Supplementary material

10.2196/102789 Multimedia Appendix 1 Patient public involvement (PPI).

10.2196/102789 Multimedia Appendix 2 Large language model selection.

10.2196/102789 Multimedia Appendix 3 Guideline-grounded rule-based algorithm.

10.2196/102789 Multimedia Appendix 4 Examples of robustness perturbations.

10.2196/102789 Multimedia Appendix 5 Robustness analysis.

10.2196/102789 Checklist 1 TRIPOD-LLM checklist.

## Abbreviations

BSG: British Society of Gastroenterology
CONSORT: Consolidated Standards of Reporting Trials
CRC: colorectal cancer
EHR: electronic health record
GPU: graphics processing unit
GSTT: Guy’s and St Thomas’ National Health Service Foundation Trust
IRAS: Integrated Research Application System
LLM: large language model
NHS: National Health Service
SWB: Sandwell and West Birmingham National Health Service Trust
TRIPOD-LLM: Transparent Reporting of a Multivariable Prediction Model for Individual Prognosis or Diagnosis–Large Language Models

## References

[1] Rutter MD, East J, Rees CJ (2020). British Society of Gastroenterology/Association of Coloproctology of Great Britain and Ireland/Public Health England post-polypectomy and post-colorectal cancer resection surveillance guidelines. Gut.

[2] Atkin W, Wooldrage K, Brenner A (2017). Adenoma surveillance and colorectal cancer incidence: a retrospective, multicentre, cohort study. Lancet Oncol.

[3] Atkin W, Brenner A, Martin J (2017). The clinical effectiveness of different surveillance strategies to prevent colorectal cancer in people with intermediate-grade colorectal adenomas: a retrospective cohort analysis, and psychological and economic evaluations. Health Technol Assess.

[4] Huang Y, Gong W, Su B (2010). Recurrence and surveillance of colorectal adenoma after polypectomy in a southern Chinese population. J Gastroenterol.

[5] Cross AJ, Robbins EC, Pack K (2021). Colorectal cancer risk following polypectomy in a multicentre, retrospective, cohort study: an evaluation of the 2020 UK post-polypectomy surveillance guidelines. Gut.

[6] Lee TJ, Siau K, Esmaily S (2019). Development of a national automated endoscopy database: the United Kingdom National Endoscopy Database (NED). United European Gastroenterol J.

[7] Ali M, Heyes A, Stephen A, Ng S, Hanif Z (2022). TU2.5 Compliance of colonic polyp surveillance with British Society of Gastroenterology (BSG) and the Association of Coloproctology of Great Britain and Ireland (ACPGBI) guidelines. BJS.

[8] Butler DM, Myintmo A, Flashman H, Ukoumunne OC, Bethune R (2023). Adherence to post-polypectomy surveillance guidelines at a large district general hospital. Cureus.

[9] Liu MC, Kassim G, Mead-Harvey C, Anderson LR, Ramirez F, Gurudu S (2023). S209 Non-adherence to colorectal polyp surveillance guidelines: magnitude and factors associated with non-adherence in an open access colonoscopy screening program at an academic institution. Am J Gastroenterol.

[10] Seong D, Choi YH, Shin SY, Yi BK (2023). Deep learning approach to detection of colonoscopic information from unstructured reports. BMC Med Inform Decis Mak.

[11] Bae JH, Han HW, Yang SY (2022). Natural language processing for assessing quality indicators in free-text colonoscopy and pathology reports: development and usability study. JMIR Med Inform.

[12] Galoosian A, Soroudi C, Jain S (2021). S259 Validation of a machine learning algorithm to measure adenoma detection rate from screening colonoscopies in a large health system. Am J Gastroenterol.

[13] Peterson E, May FP, Kachikian O (2021). Automated identification and assignment of colonoscopy surveillance recommendations for individuals with colorectal polyps. Gastrointest Endosc.

[14] Wu L, Shi C, Li J (2023). Development and evaluation of a surveillance system for follow-up after colorectal polypectomy. JAMA Netw Open.

[15] Brennan G, Osikoya OE (2023). S1702 Comparing large language models accuracy in following interval surveillance colonoscopy guidelines. Am J Gastroenterol.

[16] Acharya V, Kumaresan V, England J, Mehta S, Sussman D, Deshpande A (2025). Use of large language models to identify surveillance colonoscopy intervals-a feasibility study. Gastroenterology.

[17] Acharya V, Mehta SJ, Sussman DA (2026). Use of large language models to determine the surveillance colonoscopy interval: a bi-institutional validation study. Am J Gastroenterol.

[18] Gallifant J, Afshar M, Ameen S (2025). The TRIPOD-LLM reporting guideline for studies using large language models. Nat Med.

[19] Zeki SS (2018). EndoMineR for the extraction of endoscopic and associated pathology data from medical reports. J Open Source Softw.

[20] National data opt-out. NHS England.

[21] Abdin M, Aneja J, Behl H (2024). Phi-4 technical report. arXiv.

[22] Zhang Z, You W, Wu T, Wang X, Li J, Zhang M (2025). Proceedings of the 31st International Conference on Computational Linguistics.

[23] Djinbachian R, Khellaf A, Noyon B, Soucy G, Nguyen BN, von Renteln D (2023). Accuracy of measuring colorectal polyp size in pathology: a prospective study. Gut.

[24] Bedi S, Liu Y, Orr-Ewing L (2025). Testing and evaluation of health care applications of large language models: a systematic review. JAMA.

[25] Abbasian M, Khatibi E, Azimi I (2024). Foundation metrics for evaluating effectiveness of healthcare conversations powered by generative AI. NPJ Digit Med.

[26] Huang Y, Tang K, Chen M, Wang B (2024). A comprehensive survey on evaluating large language model applications in the medical industry. arXiv.

[27] Singh A, Singh N, Vatsal S (2024). Robustness of large language models to perturbations in text. arXiv.

[28] Moradi M, Samwald M (2021). Proceedings of the 2021 Conference on Empirical Methods in Natural Language Processing.

[29] Dong G, Zhao J, Hui T (2023). Natural Language Processing and Chinese Computing.

