# Supplementary material for: Improving Postpolypectomy Surveillance Decisions Using an Automated Analysis of Colonoscopy and Histopathology Reports: 2-Center Validation Study
Source: J Med Internet Res. 2026 Oct 16;28:e102789. doi: 10.2196/102789 (PMC13674522; doi:10.2196/102789)
Supplement: Supplementary file 1 [file jmir-v28-e102789-s001.docx]

# Patient Public Involvement (PPI)

Patient and Public Involvement was prioritised during the scoping and initial development phase of the project to ensure the acceptability and usability of our application by patients. The research questions and outcome measures were developed with an understanding of patients’ priorities and experiences gathered through in-house forums, social media engagement via Twitter, Facebook and Q&A sessions as well as feedback collected from posters placed
