# Supplementary material for: Improving Postpolypectomy Surveillance Decisions Using an Automated Analysis of Colonoscopy and Histopathology Reports: 2-Center Validation Study
Source: J Med Internet Res. 2026 Oct 16;28:e102789. doi: 10.2196/102789 (PMC13674522; doi:10.2196/102789)
Supplement: Supplementary file 2 [file jmir-v28-e102789-s002.docx]

**Large language model selection**

Comparison of open-weight instruction-tuned large language models for colorectal surveillance interval classification on the Guy’s and St Thomas’ NHS Foundation Trust test set (n=100) using identical prompting and decoding strategies. GPU memory use is reported to assess deployment feasibility.

| **Model** | **Params (B)** | **GPU memory (GB)** | **F1: no surveillance** | **F1: 3-year surveillance** | **F1: site check** | **F1: ALERT (CRC)** |
| --- | --- | --- | --- | --- | --- | --- |
| Phi-4 | 14.0 | 3.51 | 0.98 | 0.86 | 0.93 | 1.00 |
| Phi-4-mini-instruct | 3.8 | 1.05 | 0.49 | 0.26 | 0.42 | 0.89 |
| Llama-3.1-8B-Instruct | 8.0 | 1.16 | 0.89 | 0.48 | 0.69 | 1.00 |
| Falcon3-7B-Instruct | 7.0 | 1.03 | 0.89 | 0.50 | 0.79 | 1.00 |
| Mistral-7B-Instruct-v0.3 | 7.0 | 0.86 | 0.48 | 0.23 | 0.42 | 0.89 |
