# Supplementary material for: Improving Postpolypectomy Surveillance Decisions Using an Automated Analysis of Colonoscopy and Histopathology Reports: 2-Center Validation Study
Source: J Med Internet Res. 2026 Oct 16;28:e102789. doi: 10.2196/102789 (PMC13674522; doi:10.2196/102789)
Supplement: Supplementary file 3 [file jmir-v28-e102789-s003.docx]

**Guideline-grounded rule-based algorithm**

**Algorithm 1. Guideline-grounded surveillance decision using LLM-extracted polyp features.**

```
Input: Pots P (LLM output) and patient age age.
Output: Recommendation rec, premalignant count N_prem, advanced count N_adv.

N_prem, N_adv, N_polyps <- 0
needsSiteCheck, hasLNPCP_R0 <- false

for each pot in P:
  if IsCancer(pot):
    return "ALERT (colorectal cancer detected)", N_prem, N_adv

  if IsOtherPolypsSection(pot):
    N_polyps <- N_polyps + CountOtherPolyps(pot)
    needsSiteCheck <- update using MaxSizeMm(pot)
    continue

  if TypeOfSpecimen(pot) != "polyp":
    continue

  sizes <- EffectiveSpecimenSizesMm(pot)   # one size per specimen
  N_polyps <- N_polyps + len(sizes)

  update (needsSiteCheck, hasLNPCP_R0) using LNPCP rules
  (size >= 10 mm and excision status)

  for each size in sizes:
    if not IsPremalignantPolyp(pot, size):
      continue
    N_prem <- N_prem + 1
    if IsAdvancedPolyp(pot, size):
      N_adv <- N_adv + 1

if N_polyps > 10:
  return "ALERT: >10 polyps detected", N_prem, N_adv

highRisk <- IsHighRisk(hasLNPCP_R0, N_prem, N_adv)

if needsSiteCheck:
  rec <- "Site check at 2-6 months, then repeat at 12 months"
else:
  if age known and age > 75 and highRisk:
    rec <- "Consider age: patient >75 with high-risk findings"
  else if highRisk:
    rec <- "One-off surveillance in 3 years"
  else if age known and age < 45 and N_prem > 0:
    rec <- "Follow-up in 5 years (low-risk findings, age <45)"
  else:
    rec <- "No further colonoscopic surveillance; advise routine bowel screening"

return rec, N_prem, N_adv
```
