# Supplementary material for: Improving Postpolypectomy Surveillance Decisions Using an Automated Analysis of Colonoscopy and Histopathology Reports: 2-Center Validation Study
Source: J Med Internet Res. 2026 Oct 16;28:e102789. doi: 10.2196/102789 (PMC13674522; doi:10.2196/102789)
Supplement: Supplementary file 4 [file jmir-v28-e102789-s004.docx]

**Example of robustness perturbations**

Example robustness perturbations applied to a synthetic histopathology report input. Perturbations include adjacent letter swaps, character insertions and deletions, random word splits, letter case alterations.

| **Original input** | **Perturbed input** |
| --- | --- |
| Macroscopic description: specimen a- Single sessile polyp, 8x3x4mm, pale, in the sigmoid colon. specimen b-Single pedunculated polyp 12mm in the ascending colon.  Microscopic description: a) Fragments of tubular adenoma with low-grade dysplasia, excised with clear margins (R0). b) Fragments of tubulovillous adenoma. no high-grade dysplasia or carcinoma. | Macrosopic descrption: spceimen a- Singel sesile polypp, 8x3x4 mm, pale, in the sigmoi d colon. Specimen b- Single peduncualted polyp 12m m in the ascnding colon.  Microscopic description: a) Fragmetns of tubluar adenoma with lo w-grade dysplaisa, excised with clea r margins (R0). b) Framgents of tubulovillous adneoma. No high-grade dysplaisa or carci noma. |
