# Supplementary material for: Improving Postpolypectomy Surveillance Decisions Using an Automated Analysis of Colonoscopy and Histopathology Reports: 2-Center Validation Study
Source: J Med Internet Res. 2026 Oct 16;28:e102789. doi: 10.2196/102789 (PMC13674522; doi:10.2196/102789)
Supplement: Supplementary file 5 [file jmir-v28-e102789-s005.docx]

**Robustness analysis**

Robustness of the surveillance recommendation to typographical noise. Macro-averaged precision, recall, F1-score across the surveillance on the Guy's and St Thomas' NHS Foundation Trust test set (n=100) for the original (clean) reports and for corrupted versions (1 to 5 perturbations per report, CORR1, CORR3, CORR5).

| **Setting** | **Macro precision** | **Macro recall** | **Macro F1-score** |
| --- | --- | --- | --- |
| Original (clean) | 0.93 | 0.96 | 0.94 |
| CORR1 | 0.93 | 0.95 | 0.94 |
| CORR3 | 0.91 | 0.95 | 0.92 |
| CORR5 | 0.88 | 0.82 | 0.84 |
